# Expression of Potential Dermal Progenitor Cell Markers in the Tumour and Stroma of Skin Adnexal Malignant and Benign Tumours

**DOI:** 10.1155/2019/9320701

**Published:** 2019-04-01

**Authors:** Sven R. Quist, Maximilian Eckardt, André Kriesche, Harald P. Gollnick

**Affiliations:** ^1^Department of Dermatology and Venereology, Otto-von-Guericke University, Leipziger Strasse, 39120 Magdeburg, Germany; ^2^Dermatology Center MDZ, Mainz, Germany

## Abstract

Stem cells are multipotent cells that maintain the skin epidermis including skin appendages such as hair follicle, sebaceous glands, and sweat glands. There is evidence that reciprocal signalling between the epidermis and the dermis plays an important role in skin development, homeostasis, wound repair, and skin cancer. The origin of skin cancer that derive from skin appendages is still controversial, including basal cell carcinoma and even more of rare tumours such as sebaceous carcinomas and whether those tumours originate from resident tissue stem cells. To investigate whether markers reported to label dermal progenitor cells are preserved in the tumour including the tumour stroma of skin adnexal tumours, we tested 45 human basal cell carcinomas, including superficial, nodular, adenoid, infiltrating, and sclerosing types, and further 38 human tumours of skin appendages including 13 sebaceous adenomas and carcinomas, 20 eccrine sweat gland tumours, and 5 pilomatricomas, syringomas, and hair follicle tumours for the expression of the potential dermal and epidermal cell markers CRABP1, Nestin, and Ephrin B2 and compared these findings with healthy, age-related human epidermis. We detected that CRABP1, Nestin, and Ephrin B2 are expressed in the intratumoural stroma as well as the tumour invasive front of skin tumours of appendages and BCCs.

## 1. Introduction

The skin is the outermost layer of the human body, and it protects from physical or biological harm. It is a multilayer epithelium, which contains the interfollicular epidermis and adnexal structures such as the hair follicle, sebaceous glands, or sweat glands [1]. The hair follicle is a heterogeneous compartment that is believed to contain a reservoir of various stem cells capable of differentiating into different lineages such as the interfollicular epidermis or the sebaceous gland that arises from a common “pilosebaceous unit” [2]. The skin tumour stroma is part of the tumour microenvironment comprising all tissue components associated with a skin cancer that can have both tumour-inhibitory and -promoting effects. There is increasing evidence that the dermal compartment located beyond the epidermis and around the pilosebaceous unit interacts with epidermal cells in reciprocal signalling and plays an important role in skin cancer development [3, 4]. For this study, we have selected three markers: CRABP1, Nestin, and Ephrin B2, to test whether they are expressed in tumours or tumour stroma of skin adnexal tumours since it has been reported previously that they are (a) involved in human embryology and development of the epidermal and especially dermal compartment and (b) expressed in skin cancer.

Lineage-tracing experiments have identified that although retinoic acid (RA) signalling is essential for epidermal differentiation, the RA-binding protein CRABP1 is dynamically expressed in the embryonic dermis as well as in the stroma of skin tumours [5] and plays a role in malignant transformation of mesenchymal cells [6]. The lifetime risk of many cancers strongly correlates with the total number of divisions of the stem cells that maintain tissue's homeostasis [7]. Furthermore, CRABP1 together with *β*-catenin was expressed in sebaceous gland tumours, and CRABP1 as part of retinoic acid signalling enhanced malignancy of human mesenchymal cells [6] and invasiveness of oral squamous cell carcinoma in vitro [8, 9]. Nestin is an intermediate filament protein expressed by migrating and proliferating neural crest stem cells during their embryogenesis [10]. It is regarded as a biomarker of multilineage progenitor cells, and its expression may indicate cell pluripotency and regeneration [11]. In the human skin, nestin expression has been reported in hair follicle progenitor cells that differentiate into adipocytes, fibrocytes, or neurons [10, 12]. In previous experiments, the stroma of trichoblastomas contained nestin-positive cells, but the stroma of the nevus sebaceous or basal cell carcinomas was negative for nestin [13]. Erythropoietin-producing hepatocellular (Eph) receptor tyrosine kinases (RTKs) are activated upon binding to their membrane-associated ephrin ligands [14]. Eph receptors and their membrane-bound ephrin ligands play a role in a wide variety of embryonic processes including the skin [14, 15]. Mesenchymal stromal/stem cells (MSC) express the contact-dependent erythropoietin-producing hepatocellular (Eph) receptor tyrosine kinase family and their cognate ephrin ligands, which are known to regulate thymocyte maturation and selection, T-cell transendothelial migration, activation, costimulation, and proliferation [16–20]. Ephrin-B2 is expressed by ex vivo expanded MSC and ADAM10-mediated sEphrin-B2 generation that is required for TGF-*β*1-induced myofibroblast activation [16]. Furthermore, EphB2 plays a role in the progression of cutaneous squamous cell carcinoma. EphB2 is specifically overexpressed by cutaneous squamous cell carcinoma cells and promotes proliferation, migration, invasion, and growth of this tumour [21]. In this study, we analysed two different markers for EphB2, one binding to the corresponding amino acids 1036-1051 of Human Ephrin, the C-terminal epitope and the other one to amino acids 118-133 of Human Ephrin B2, the N-terminal epitope as we were expecting to label two different mesenchymal cell types potentially involved in the formation of the tumour stroma.

In this study, we addressed the question whether different regulators of the dermal compartment such as CRABP1, Nestin, and Ephrin B2 are markers preserved directly in the surrounding tumour stroma of skin appendages tumours. In the last years, experiments have shown that cells are capable of exhibiting plasticity and changing fate through dedifferentiation and transdifferentiation upon reciprocal signalling between the epidermis and the dermis. Dermal responses to skin appendage tumorigenesis reflect a combination of changes leading to different microenvironments that might be driven by multilineage progenitor cells of the mesenchymal compartment. To ensure that these cells are part of the compartment of the cancer-associated stroma, we performed Herovici's collagen stain to differentiate between young newly generated collagen of the tumour stroma and mature collagen that represent the unaffected dermal compartment. We also tried to address the question whether they are associated with an either well or poorly differentiated skin tumour state. We have shown before, that within cells of the most frequent nonmelanoma skin cancer basal cell carcinoma, and other adnexal benign or malignant proliferations such as sebaceous adenoma or carcinoma and eccrine sweat gland tumours such as sebaceous carcinomas and porocarcinomas, stem cell markers such as Lrig1-, Lgr5-, Sox9 are preserved.

In order to test whether several markers reported to be important in the dermal and epidermal embryonic and postnatal development, such as CRAPB1, Nestin, Ephrin B2C (C-terminus marker), and B2N (N-terminus marker), are conserved in various human tumours of skin appendages, the number of cells, amount of expression, and localization of these markers were analysed in 45 human basal cell carcinomas (BCC, superficial, nodular, adenoid, infiltrating, and sclerosing types), 13 sebaceous adenomas and carcinomas, 20 eccrine sweat gland tumours, and 5 pilomatricomas.

## 2. Methods

We used the sections from paraffin-embedded material of skin tumours that were taken with informed consent and stored at the DermPath Laboratory of the Department of Dermatology, Otto-von-Guericke University Magdeburg, and used for staining in accordance with the recommendations of the IRB and GMC. Sections were deparaffinised, and antigen retrieval was used with either citrate buffer pH 6 (Nestin) or EDTA pH 9 (CRAPB1, Ephrin B2N, and B2C). Primary antibodies Nestin (Abcam, ab22035 1 : 300), CRAPB1 (Abcam, ab2816, 1 : 1000), Ephrin B2-N-terminus corresponding to amino acids 118-133 of Human Ephrin B2 (Epitomics, T2599 (later Abcam ab5418), 1 : 50), and B2-C-terminus corresponding to amino acids 1036-1051 of Human Ephrin B2 (Epitomics, T2600, later Abcam, ab5425, 1 : 50) were incubated for 3 h, and Zymed histostain plus kit using AEC (red colour; Zytomed) was utilized for detection and counterstained with hematoxylin. In addition to differentiating between the tumour and the stroma, we performed Herovici's collagen stain (Herovici's Collagen Stain Kit, American MasterTech, Solution A and B according to the manufacturer's protocol) to identify young newly generated collagen of the tumour stroma and mature collagen that represent the unaffected dermal compartment.

**Figure 1:**
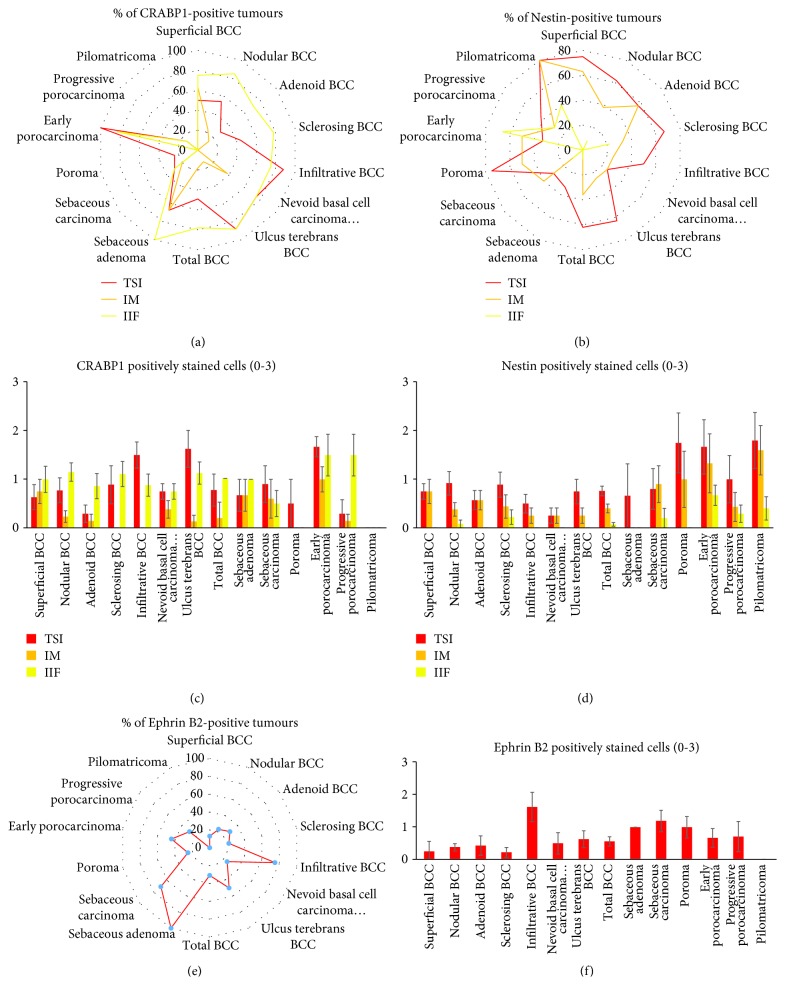
Percentage of positive tumours for CRABP1, Nestin, and Ephrin B2. Percentage of CRABP1- and Nestin-positive tumours for invasive margin (IM) of the tumour, the inflammatory infiltrate surrounding the tumour (IIF), and the tumour-stroma interaction (TSI) site within the core of the tumour (a and b). CRABP1 and Nestin positively stained cells classified into staining for positive tumours were classified into 4 groups: 0 (no expression), 1 (0-33% of cells positive), 2 (33-66% of cells positive), and 3 (>66% of cells positive, mean with standard deviation, c and d). Ephrin B2-positive tumours in % (e) and classified staining (mean with standard deviation) (f).

**Figure 2:**
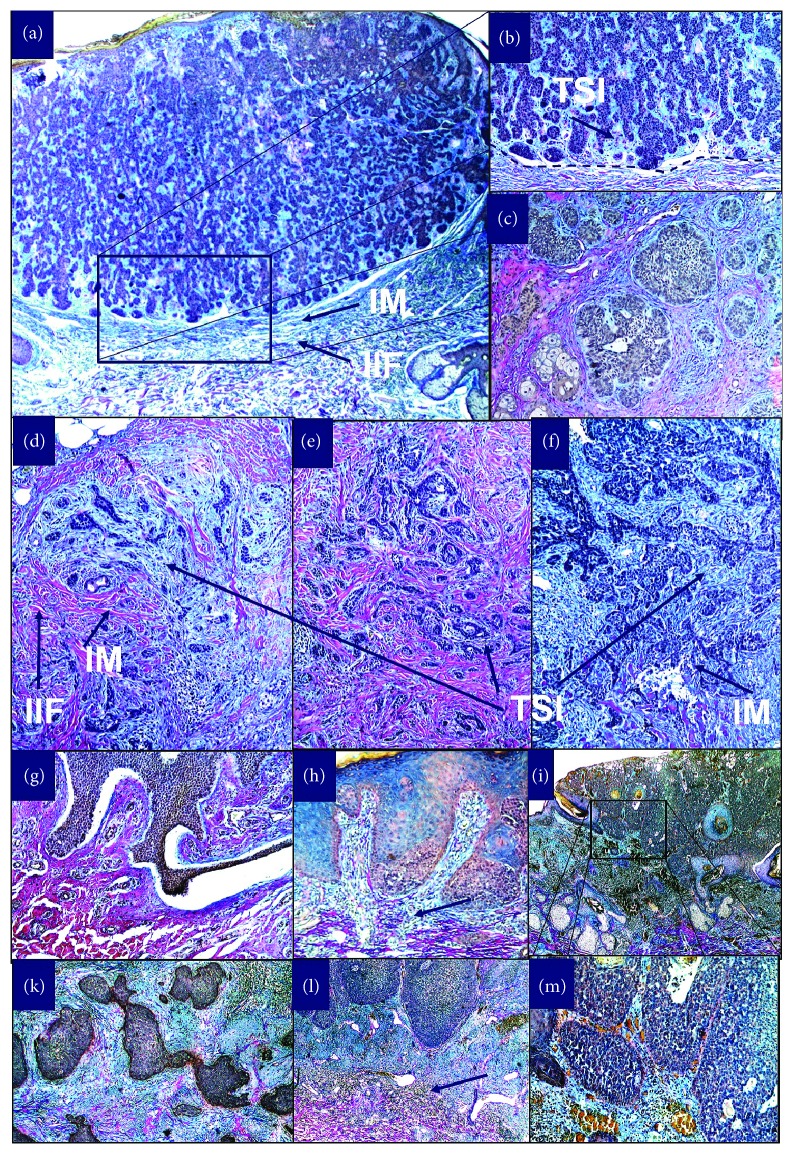
Herovici's stain of skin appendage tumours: stain of young (blue) and mature (pink) collagen (Herovici's stain), was used to allocate tumour stroma of skin appendage tumours as indicated by young collagen. The amount of cells that are present at either the invasive margin (IM) of the tumour, the inflammatory infiltrate surrounding the tumour (IIF), or the tumour-stroma interaction (TSI) site within the core of the tumour were determined for each tumour. Nodular basal cell carcinoma with stroma (blue indicating young collagen) within the tumour's stroma interface and at the tumour's invasive margin surrounded by its inflammatory interface (a, b). Adenoid basal cell carcinoma with blue stroma within the tumour surrounded by pink, mature collagen (c). Different stains of sclerosing basal carcinoma: blue young collagen largely present (d, f), almost absent (e) within the tumour and indicating the tumour margins (d) or invasive front (f) where tumour cells invade mature, pink collagen. Poroma with only mature (pink) collagen (g) in contrast to early porocarcinoma (h, l) with mixed blue and pink stroma and a margin of blue and pink (black arrow) and progressive porocarcinoma (l) with dominance of blue collagen with single fibers of pink collagen. Sebaceous carcinoma with tumour stroma as indicated by blue stained collagen ((i) and (m) with higher magnification).

Skin sections were evaluated by 2 independent dermatohistopathologists for the markers tested. First, normal epidermis and hair follicles or skin appendages were evaluated, and expression in different anatomical important areas was noticed (Table 1). Evaluation of tumours was done in different ways. First, the staining for positive tumours was classified into 4 groups: 0 (no expression), 1 (0-33% of cells positive), 2 (33-66% of cells positive), and 3 (>66% of cells positive). Then, the average was calculated from all tumours within a specific subgroup (±standard error). Next, the percentage of positive tumours for each marker was evaluated (Figures 1(a), 1(b), and 1(e)). Finally, for two markers CRABP1 and Nestin that were predominantly present in the stroma of skin tumours, we used Herovici's collagen (Figure 2) stain to allocate the expression profile of these markers and slide; we analysed the amount of cells that are present at either the invasive margin (IM) of the tumour, the inflammatory infiltrate surrounding the tumour (IIF), and the tumour-stroma interaction (TSI) site within the core of the tumour (Figures 1(c) and 1(d)), a method that has been established in the analysis of gastrointestinal tumours [22, 23]. For Ephrin B2, since expression was noticed in the tumour cells and stroma, we evaluated the percentage of positive tumours and cells according to the 4 groups: 0 (no expression), 1 (0-33% of cells positive), 2 (33-66% of cells positive), and 3 (>66% of cells positive, Figures 1(e) and 1(f)).

## 3. Results

### 3.1. CRABP1

**Figure 3:**
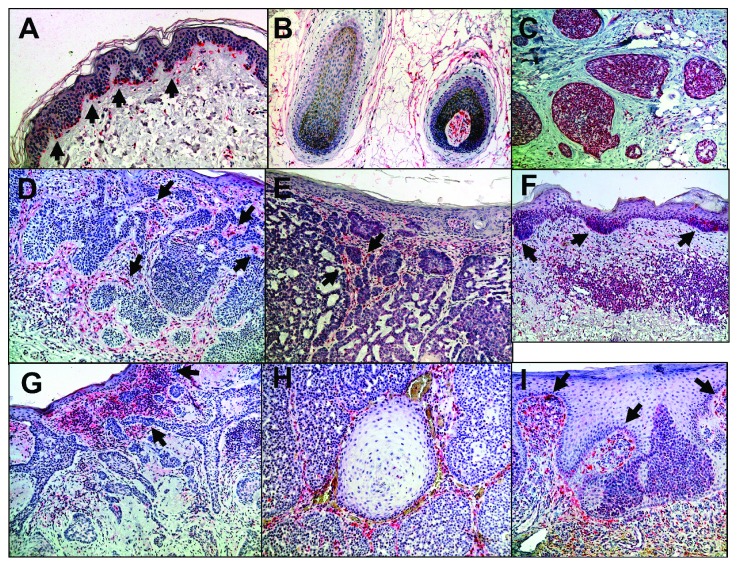
CRAPB1 expression profile: positive staining of cells lining up at the epidermal-dermal interface region and the papillary dermis ((a), black arrows) and the dermal papilla (b) and strong staining of sebaceous gland cells, their septates and within the surrounding papillary dermis (c). Nodular basal cell carcinoma with staining at the tumour-stroma interaction (TSI) site within the core of the tumour (as indicated by black arrows, (d)) but also within the tumour (e). Superficial basal cell carcinoma with CRABP1-positive cells at the tumour-stroma interaction site ((f), black arrows). Sclerosing basal cell carcinoma with strong expression within the inflammatory infiltrate located within the core of the tumour and its tumour-stroma interaction (TSI) site (g). Well-differentiated sebaceous carcinoma (h) with positive cells located at the tumour-stroma interaction (TSI) site within the core of the tumour and at the invasive margin (IM) of the tumour in an early porocarcinoma (i).

In unaffected skin, CRABP1 staining was detected at the epidermal-dermal interface and the papillary dermis (Figure 3(a)) where the cells were lining at the epidermal-dermal interface and around the dermal papilla (Figure 3(b)). Strong staining of sebaceous gland cells, their septates, and within the surrounding papillary dermis was observed (Figure 3(c)). The nodular basal cell carcinoma with staining at the tumour-stroma interaction (TSI) site not only within the core of the tumour but also within the tumour stroma itself is shown in Figure 3(d) (as indicated by black arrows) and Figure 3(e), respectively. In superficial basal cell carcinomas, we observed CRABP1-positive cells at the tumour-stroma interaction site, when the tumour was in the probes of the formation and early invasion (Figure 3(f)). Sclerosing basal cell carcinoma with strong expression within the inflammatory infiltrate located within the core of the tumour and its tumour-stroma interaction site (TSI, Figure 3(g)). Well-differentiated sebaceous carcinoma is shown in Figure 3(h) with positive cells located at the tumour-stroma interaction (TSI) site within the core of the tumour and at the invasive margin (IM) of the tumour in an early porocarcinoma (Figure 3(i)). The expression of CRABP1 was found in the IM in the early superficial BCCs as well as in the TSI and IIF of more aggressive BCC including sclerosing and infiltrative types where up to 88% of tumour stroma was positive for CRABP1 (Figure 1(c)). In sebaceous tumours, CRABP1 was found more expressed in adenomas than in carcinomas and in early well-differentiated porocarcinoma than in more aggressive and progressed porocarcinoma (Figure 1(a)).

### 3.2. Nestin

**Figure 4:**
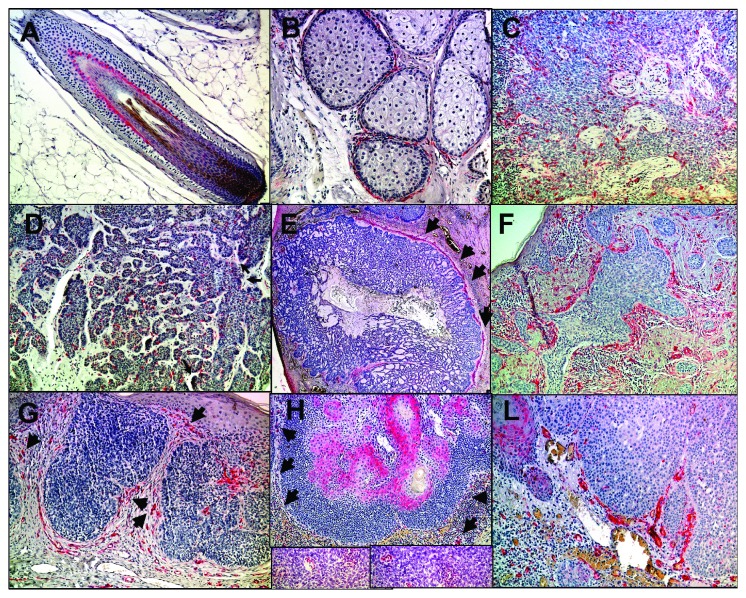
Nestin expression profile: scalp skin with positive staining of the outer root sheath cells of the hair follicle (a) in the dermal interface of sebaceous glands (b) and the tumour-stroma interaction (TSI) site within the core of the tumour and the invasive margin (IM) of the tumour of a nodular basal cell carcinoma (c, d), adenoid (e), and nodular (f) basal cell carcinoma with Nestin-positive cells at the invasive margin (IM) of the tumour (black arrows). Expression in early porocarcinoma at the invasive margin (g and h) but also within the inflammatory infiltrate ((h), as indicated by black arrows and the two small pictures below in higher magnification). Low-grade sebaceous carcinoma (i) with positive basal cells within the tumour stroma interface (TSI).

In unaffected skin of the scalp, Nestin-positive cells are located at the outer root sheath cells of the hair follicle (Figure 4(a), Table 1) in the dermal interface of sebaceous glands (Figure 4(b)) and the tumour-stroma interaction site within the core of the tumour (TSI) as well as the invasive margin (IM) of the tumour of a nodular basal cell carcinoma (Figures 4(c) and 4(d)). Nestin-positive cells were located at the invasive margin (IM) of the tumour of an adenoid basal cell carcinoma (Figure 4(e)). Sebaceous carcinoma with positive cells at the invasive margin (IM) of the tumour is shown in Figure 4(f). Nodular basal cell carcinoma with positive dendritic-shaped cells at the tumour-stroma interface (TSI ) is shown in Figures 4(c) and 4(d)). Adenoid (Figure 4(e)) and nodular (Figure 4(f)) basal cell carcinoma presented Nestin-positive cells at the invasive margin (IM) of the tumour. Expression of Nestin-positive cells within an early porocarcinoma at the invasive margin (Figures 3(g) and (Figure 3(h)) belonged to not only the tumour stroma as verified by Herovici's stain (Figure 2(g)) but also within the inflammatory infiltrate (Figure 3(h)). Low-grade sebaceous carcinoma (Figure 4(i)) showed positive basal cells within the tumour stroma interface. Nestin was expressed in the TSI of all skin adnexal tumours. Expression was higher in low-grade tumours such as superficial BCC or sebaceous adenoma or poroma within the TSI or IM and less expressed in the TSI or IM in advanced skin tumours such as sclerosing or infiltrative BCC, sebaceous carcinoma, or progressive porocarcinoma (Figure 1(d)). In contrast, it was less expressed within the IIF of skin appendage tumours than within the TSI or IM with a similar trend to be downregulated in more advanced skin tumour types (Figures 1(b) and 1(d)).

### 3.3. Ephrin B2

**Figure 5:**
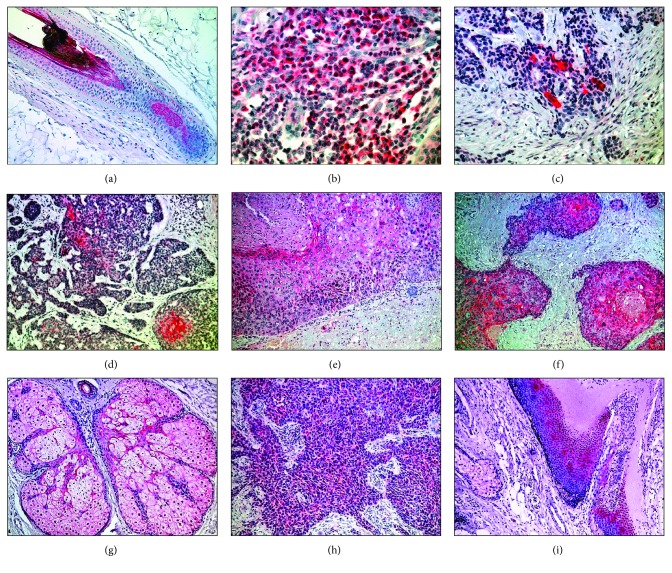
Ephrin B2C expression profile: hair follicle with precortical (black arrow) and inner root sheath staining (a). Sclerosing BCC with positive cells at the tumour-stroma interaction (TSI) site within the core of the tumour together with plasma cells, infiltrating and surrounding BCCs as well (b). Infiltrating BCC (c) and adenoid BCC (d) are mainly negative except for a few cells at the tumour-stroma interaction (TSI) site. Early porocarcinoma (e) and late porocarcinoma showed both expressions (f). Similar results for sebaceous tumours: sebaceous adenoma (g) is weakly positive in contrast to an undifferentiated sebaceous gland carcinoma (h). Pilomatricoma with positive transitory cells (i).

**Figure 6:**
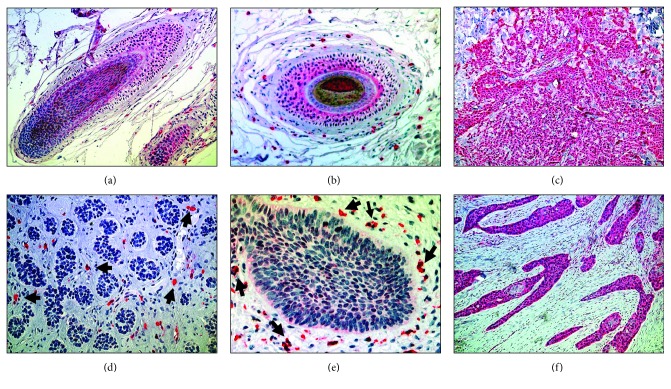
Ephrin B2N expression profile: in unaffected skin, the hair follicle shows positive staining of the inner root sheath and bulbus (a and b). Cells of the hair follicle showed strong cytoplasmic with weaker nuclear staining (c). Adenoid BCC with positive cells at the tumour-stroma interaction (TSI) site within the core of the tumour (black arrows) (d) and positive cells at the invasive margin (IM) of superficial BCC (e). Strong staining of a sclerosing BCC (f).

All tumours except pilomatricoma were positive for the expression of Ephrin B2 (Figures 1(e) and 1(f)). Ephrin B2 expression was detectable in the stroma of tumours but did also stain tumour cells (Figures 5 and 6). In unaffected skin, hair follicle showed expression within the precortical, the inner root sheath, and the bulbus (Figure 5(a), Figures 6(a) and 6(b)). Sclerosing BCC with positive cells at the tumour-stroma interaction (TSI) site within the core of the tumour together with plasma cells infiltrates that surrounds the BCCs as well (Figure 5(b)). Whereas Ephrin B2C stained only cells within the stroma, Ephrin B2N stained not only sclerosing BCCs (Figure 6(f)) but also 75% of infiltrative BCCs (Figures 1(e) and 1(f)). Adenoid BCCs (Figure 5(d)) were mainly negative for both Ephrin B2 markers except a few cells at the tumour-stroma interaction (TSI) site. Furthermore, positive cells were recognized at the invasive margin (IM) of superficial BCC (Figure 5(d)). Early porocarcinoma (Figure 5(e)) and late porocarcinoma (Figure 5(f)) showed both a few tumours positive for Ephrin B2C with strongest expression in the more advanced progressive porocarcinoma. Sebaceous adenoma (Figure 5(g)) showed weak expression in contrast to an undifferentiated sebaceous gland carcinoma (Figure 5(h)). Pilomatricoma with positive transitory cells is shown in Figure 5(i). Adenoid BCC showed positive cells at the tumour-stroma interaction site within the core of the tumour for Ephrin B2N (Figure 6(d)) and positive cells at the invasive margin (IM) of superficial BCC (Figure 6(e)). Strong staining was observed for a sclerosing BCC with Ephrin B2N (Figure 6(f)). Both markers, Ephrin B2C and Ephrin B2N, label cells in the dermis, within the tumour stroma and tumour cells of BCCs but only Ephrin B2C is expressed in the porocarcinomas and sebaceous gland tumours.

## 4. Discussion

In addition to the long debate on the origin of tumours that are derived from the skin appendages of basal cell carcinoma, the most frequent one, in particular, we have previously reported that Lgr5, Lrig1, and cytokeratin 15 were expressed in all skin appendage tumours tested, and expression of Lgr5 and Lrig1 was generally lower expressed in more aggressive tumour types such as sclerosing basal cell carcinoma and late porocarcinoma stage than in less aggressive superficial or nodular basal cell carcinoma or early porocarcinoma and sebaceous gland tumours [24]. In this study, we have addressed whether the markers that are reported to be potential dermal progenitors in the literature are also expressed in the tumour stroma or within the tumour itself in the tumours of the skin appendages.

In previous studies, CRABP1 has been detected in murine papillomas and SCCs within the tumour stroma and at the junctions between the epidermal Keratin 14-positive cells and the dermis [5]. Furthermore, CRABP1 was overexpressed in some areas within the sebaceous carcinomas of the eyelids [8]. It has been shown before that Nestin is expressed in the hair follicle cells below the sebaceous glands and the outer-root sheath cells of the hair follicle in accordance with our results [25]. Nestin is further expressed in the tumour stroma of trichoblastomas but not BCCs [13]; other studies did not find Nestin a useful marker in the differentiation of these exact tumours as it was nonsignificantly differently expressed [26]. Nestin has been analysed together with CD133 in melanoma, SCCs, and BCCs, and when using Nestin, it was most suitable to differentiate the melanoma subtype [27]. In BCCs of this analysis, Nestin was weakly expressed in the majority of BCCs but the tumour stroma was not separately analysed. Another group reported that BCCs and Merkel cell carcinomas were negative for Nestin; however, 45% of SCC cases were Nestin positive [28]. Ephrin A has been primarily investigated and found to be overexpressed in squamous cell carcinomas (SCCs) activated by Ras signalling [29]. However, it has been reported that Ephrin B2 promotes squamous cell carcinoma as well targeting MMD1 and MMP13 [21].

In this study, we have tested for the first time the potential markers of dermal progenitor cells in a wide range of skin appendage tumours, including BCCs, as the most frequent skin cancer and sebaceous carcinomas, porocarcinomas, and pilomatricomas.

In addition to the usual analysis of immunohistochemistry staining results, we used an analysis method that has been established in the analysis and prognostic evaluation of gastrointestinal tumours. It incorporates the differences in the distribution of cells, whether the cells are allocated to the invasive margin (IM) of the tumour, the inflammatory infiltrate surrounding the tumour (IIF), or the tumour-stroma interaction (TSI) site within the core of the tumour.

CRABP1 was expressed at the IM in early formed superficial BCCs indicating a role in the formation of BCCs. It was also expressed in the TSI and IIF of more aggressive BCC including sclerosing and infiltrative types indicating its role in the progression and invasion of the tumour. Since we used Herovici's collagen stain to allocate CRABP1-positive cells in the tumour stroma and the preexisting dermis, we could verify that CRABP1-positive cells are located in the tumour stroma and located in the environment of tumour cells. In sebaceous tumours, CRABP1 was expressed in adenomas more than carcinomas and in early well-differentiated porocarcinoma more than in more aggressive and progressed porocarcinoma indicating a role in the formation and the transition of these tumours rather than in progression and invasion.

Nestin was expressed in the tumour stroma of all skin adnexal tumours. It was higher expressed within the stroma of low-grade tumours such as superficial BCC or sebaceous adenoma or poroma and less expressed in more progressed skin tumours such as sclerosing or infiltrative BCC, sebaceous carcinoma, or progressive porocarcinoma. Interestingly, Nestin main expression was observed in the stroma interacting with tumour cells (TSI) and the invasive margin (IM), indicating an active role in reciprocal signalling between the tumour and the stroma cells. In contrast, it was low expressed within the immune infiltrate surrounding the tumour indicating its role in the tumour stroma.

We further observed that Ephrin B2, a marker known to be upregulated not only in the mesenchymal compartment but also in SCCs, was expressed in the tumour stroma and within skin appendage tumours in more advanced BCCs (infiltrative BCCs and ulcus terebrans), sebaceous carcinomas, and early porocarcinomas.

In conclusion, we could detect several markers that have been reported as progenitor cells in the dermal compartment within the stroma of tumours of skin appendages indicating that they have a role in the formation of these tumours.

## Figures and Tables

**Table 1:** Expression profile of potential dermal progenitor markers in normal skin (epidermis and dermis).

Antibody		CRABP1	Nestin	Ephrin B2C	Ephrin B2N
Anatomical structure					

Epidermis	Str. corneum	0	0	0	0
	Str. granulosum	0	0	0	0
	Str. spinosum	0	0	0	0
	Str. basale	0	0	0	0

Hair follicle	Epidermal HRS	0	0	0	0
	Outer HRS	0	2	1	1
	Inner HRS	0	0	1	1

Cortex	Hair matrix	0	0	1	1
	Presumptive cortex	0	0	3	1

Sebaceous gland	Luminal	0	0	2	0

Acinus	Basal	0	0	0	0

Sebaceous gland	Basal sebocytes	1	0	0	0
	Suprabasal sebocytes	2	0	2	1

Eccrine sweat glands		0	0	0	0

Acrosyringium	Basal cells	0	0	0	0
	Luminal cells	0	0	0	0

Dermis	Epidermal/dermal interface	2	1	0	0
	Papillary dermis	1	1	1	0
	Reticulary dermis	0	1	0	0

0 = no expression detectable. 1 = <33% of cells stained positively. 2 = 33-66% of cells stained positively. 3 = >66% of cells stained positively.

## Data Availability

The data used to support the findings of this study are included within the article as figures and tables.
